# The inhibitory effect of TU-100 on hepatic stellate cell activation in the tumor microenvironment

**DOI:** 10.18632/oncotarget.27835

**Published:** 2020-12-08

**Authors:** Yuma Wada, Kazunori Tokuda, Yuji Morine, Shohei Okikawa, Shoko Yamashita, Tetsuya Ikemoto, Satoru Imura, Yu Saito, Shinichiro Yamada, Mitsuo Shimada

**Affiliations:** ^1^Department of Surgery, Graduate School of Biomedical Sciences, Tokushima University, Tokushima, Japan; ^*^These authors contributed equally to this work

**Keywords:** CRLM, TU-100, HSC, CRC, IL-6

## Abstract

Introduction: The tumor microenvironment is involved in acquiring tumor malignancies of colorectal liver metastasis (CRLM). We have reported that TU-100 (Daikenchuto) suppresses hepatic stellate cell (HSC) activation in obstructive jaundice. In this study, we report new findings as the direct and indirect inhibitory effects of TU-100 on cancer cell growth through the suppression of HSC activation.

Materials and Methods: The HSCs (LX2) were cultured in colon cancer cells (HCT116 and HT29)-conditioned medium (CM) with or without TU-100 treatment (90, 270, 900 μg/ml). Activated HSCs (aHSCs) were detected by α-SMA and IL-6 mRNA expressions and cytokine arrays of HSC’s culture supernatants. Cancer cell growth was analyzed for proliferation and migration ability, compared with TU-100 treatment. To investigate the direct anti-tumor effect of TU-100, cancer cells were cultured in the presence of aHSC-CM and TU-100 (90, 270, 900) or aHSC-CM alone, and assessed autophagosomes, conversion to LC3-II protein, and Beclin-1 mRNA expression.

Results: Colon cancer-CM significantly increased α-SMA and IL-6 mRNA expressions of aHSC. α-SMA and IL-6 mRNA expressions of aHSC, and IL-6 secretions from aHSCs were significantly decreased with TU-100 (270, 900) treatment, compared to colon cancer-CM alone. Compared with normal culture medium, aHSC-CM led to a significantly increased cell number and modified HSC-CM (TU-100; 270, 900) significantly suppressed cancer cell growth and migration. TU-100 (900) treatment induced autophagy and significantly promoted the autophagic cell death.

Conclusions: TU-100 inhibited colon cancer cell malignant potential by both suppressing HSC activation and inducing directly autophagy of cancer cells.

## INTRODUCTION

Colorectal cancer (CRC) is the second most common cause of cancer death worldwide, and the incidence is expected to increase over the next few years [1–3]. Multidisciplinary chemotherapy improves survival and becomes the standard treatments for advanced CRC [4, 5]. Additionally, herbal medicine is considered an effective resource and important in pharmacological research and drug development [6]. Many of these compounds have been used to treat a variety of diseases in clinical, even in CRC [7].

TU-100 (Daikenchuto) is a commonly prescribed Japanese herbal medicine that has been used to treat gastrointestinal motility and prevent post-operative intestinal dysfunction providing the most preclinical and clinical efficacy in several trials [8–14]. TU-100 is a mixture of herbal medicines such as processed ginger (Zingiberis Siccatum Rhizoma), ginseng (Ginseng radix), Japanese pepper (Zanthoxylum fruit), and maltose sugar by a weight ratio of 5.6%, 3.3%, 2.2%, 88.9%, respectively [10, 15]. Each compound contains specific extracts such as sanshools in Japanese pepper; ginsenosides and polysaccharides in ginseng; and gingerols and shogaols in ginger [16]. It has been reported that TU-100 has anti-inflammatory by decreasing expression of inflammatory cytokines and anti-cancer effects by inducing programmed cell death in animal model [16–21], although its mechanism is not fully known.

In the cancer microenvironment, tumor progression is associated with the interaction between cancer cells and stromal cells such as the cancer-associated fibroblast (CAF) [22–27]. It has been also well-known that in the microenvironment of colorectal liver metastasis (CRLM), hepatic stellate cells (HSCs) are activated to CAFs by cancer cells and consequently various effects of tumor malignancy such as cell proliferation, invasion and liver fibrosis are enhanced [28]. We have previously reported that TU-100 improved liver fibrosis and decreased expressions of α-smooth muscle actin (α-SMA), collagen type 1 (Colla1), and tissue inhibitor of metalloproteinase 1 (Timp1) in a rat model of common bile duct ligation through the suppression of activated HSCs [29]. Given these previous findings, the aim of this study was to investigate the anticancer effect of TU-100 through the regulation of the interaction between HSCs and cancer cells.

## RESULTS

### TU-100 inhibited HSC activation in cancer-conditioned medium

**Figure 1:**
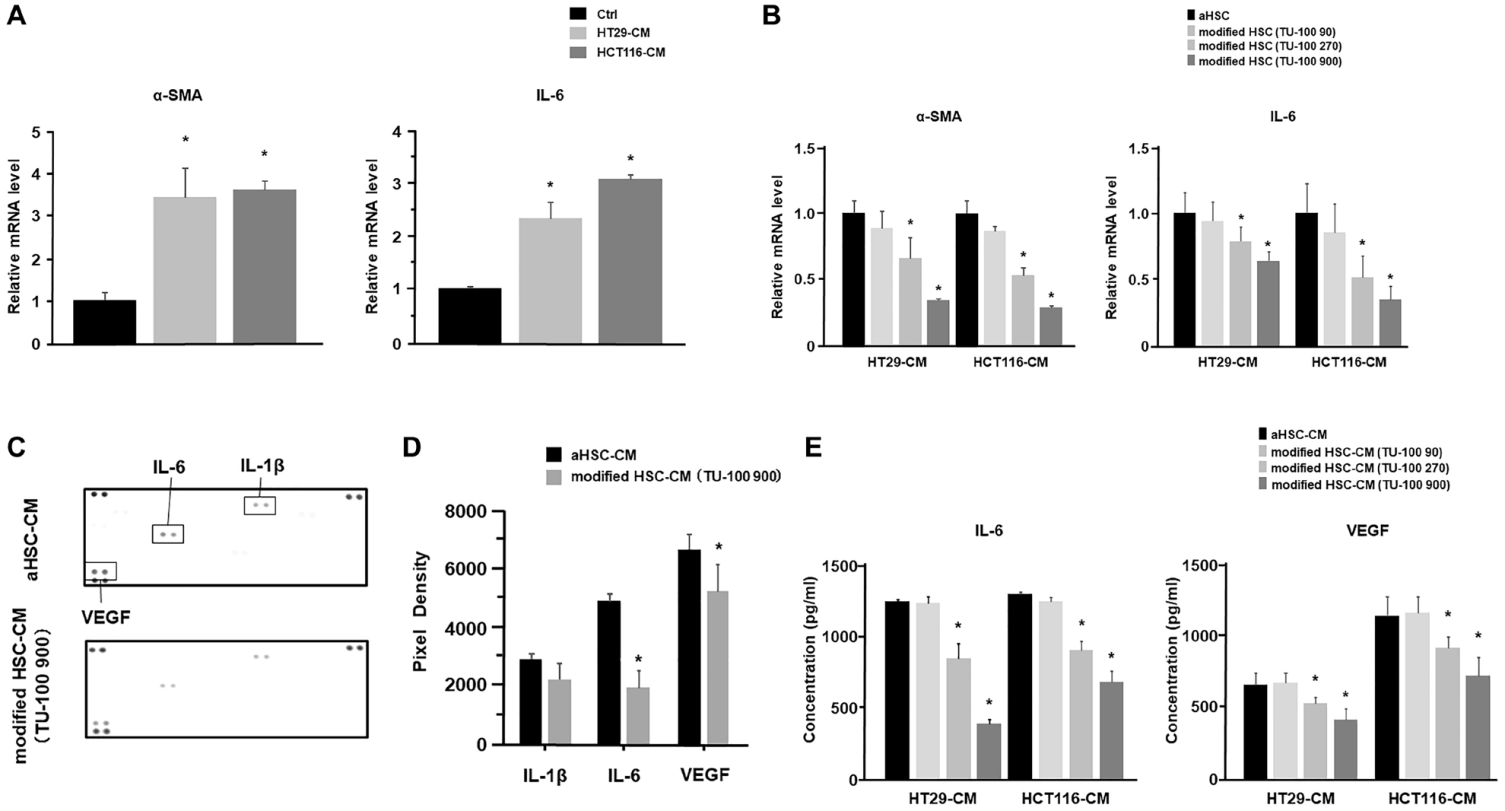
TU-100 inhibited HSC activation and cytokine’s secretions from HSCs. (**A**) HSC was stimulated with cancer-conditioned medium (cancer-CM) derived from the 24 hours cancer cells (HT29 and HCT116) culture or cultured with normal medium (control; Ctrl). α-SMA and IL-6 mRNA expressions of activated HSCs in cancer-CM were analyzed by PCR analysis. (**B**) HSCs were cultured in cancer-CM, with simultaneous TU-100 treatment: (90: 90 μg/mL; 270: 270 μg/mL; and 900: 900 μg/mL), as described in Supplementary Figure 1. α-SMA and IL-6 mRNA expressions were analyzed by PCR analysis. (**C**) Cytokine arrays of HSC’s culture supernatants (aHSC-CM) with or without TU-100 (900) treatment. (**D**) Pixel density of dot plots was calculated by using ImageJ software. (**E**) IL-6 and VEGF secretions from HSCs with or without TU-100 treatment were analyzed by ELISA. (^*^significantly different from Ctrl, *P* < .05, *n* = 4. Mann-Whitney *U* test).

At first, to investigate the effects of TU-100 on HSC activation in cancer-conditioned medium (cancer-CM) derived from 24 hours colon cancer cell culture, we assessed activated HSC (aHSC) and modified HSC (Supplementary Figure 1). Since previous studies have reported that interleukin-6 (IL-6) was a precancerous cytokine, which can promote the migration of cancer cells [30], this study also tried to check the role of IL-6 in the interaction between HSCs and cancer cells. Activated fibroblasts or myofibroblasts with α-SMA expression are the main cellular constituents of reactive stroma in some solid tumors [31]. The HSCs are one of the vital stromal component and known to be activated or transdifferentiated through intercellular communication to become myofibroblast-like cells [32], and these activated HSCs show high expression of α-SMA [33]. Results showed that aHSCs in cancer-CM significantly increased messenger RNA (mRNA) expressions of α-SMA and IL-6 (Figure 1A). Meanwhile, HSCs were cultured in cancer-CM with simultaneous TU-100 treatment (90: 90 μg/mL, 270: 270 μg/mL, and 900: 900 μg/mL), which were called as modified HSCs, and α-SMA and IL-6 mRNA expressions were significantly reduced in modified HSCs with TU-100 treatment (270, 900) (*p* < 0.05, Figure 1B).

Cytokine arrays of the HSCs culture supernatants were carried out to determine the changes in the cytokine secretion profile of HSC activation in cancer-CM according to the presence or absence of TU-100 treatment. Results showed that IL-6 and vascular endothelial growth factor (VEGF) secretions from modified HSCs with TU-100 (900) treatment were significantly decreased (*p* < 0.05, Figure 1C and 1D). In addition, the IL-6 and VEGF secretions of modified HSCs with TU-100 treatment were measured in the same dose dependent manner and it was confirmed that the TU-100 (270, 900) treatment could downregulate IL-6 and VEGF secretions from modified HSCs, compared to cancer-CM alone (Figure 1E).

### TU-100 modified HSC-CM reduced cancer cell migration and proliferation

**Figure 2:**
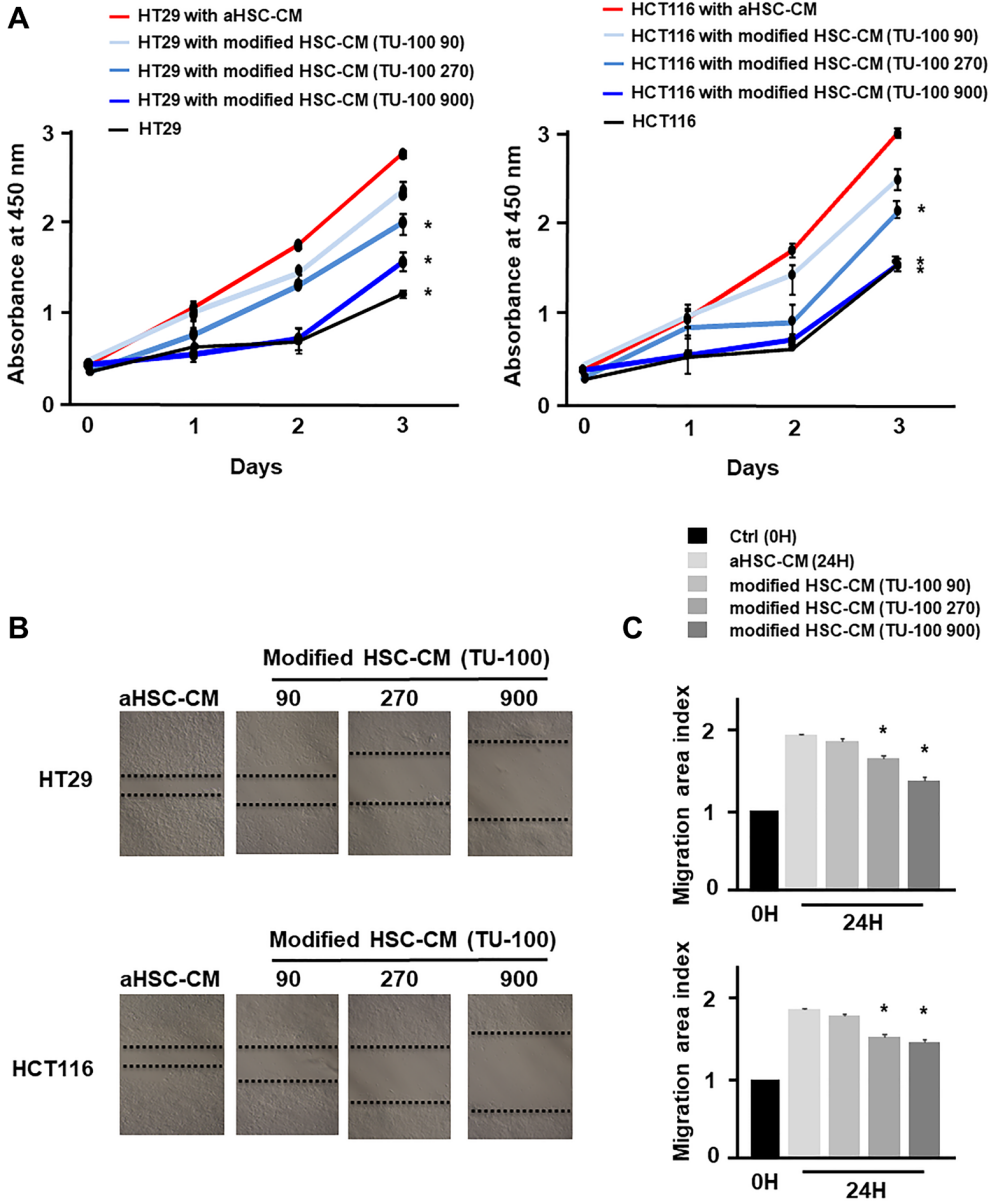
TU-100 modified HSC-CM reduced cancer cell migration and proliferation. Cancer cells (HT29 and HCT116) were cultured for 24 hours with aHSC-CM or modified HSC-CM as described in Supplementary Figure 2. (**A**) Proliferation assay and (**B** and **C**) migration assay of cancer cells were monitored for 3 days and 24 hours, respectively. (^*^significantly different from aHSC-CM group without TU-100 treatment, *P* < .05, *n* = 4, The one-way ANOVA with Turkey-Kramer’s test (proliferation assay), Mann-Whitney *U* test (migration assay).

**Figure 3:**
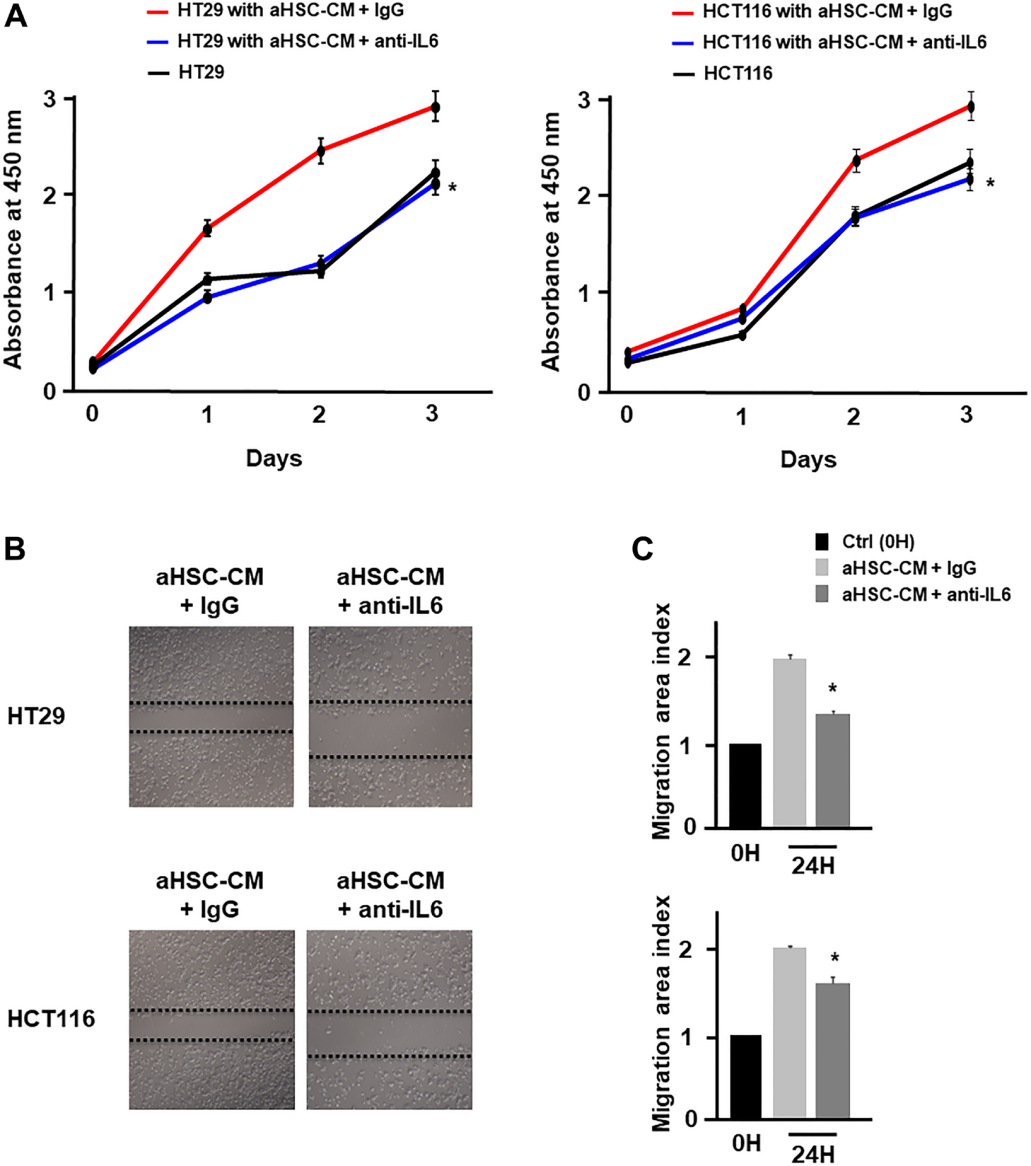
HSC derived from IL-6 promoted cancer cell proliferation and migration. (**A**) Proliferation assay and (**B** and **C**) migration assay of cancer cells in aHSC-CM with or without an IL-6 antibody were monitored for 3 days and 24 hours, respectively. (^*^significantly different from aHSC-CM group without IL-6 neutralization, *P* < .05, *n* = 4, The one-way ANOVA with Turkey-Kramer’s test (proliferation assay), Mann-Whitney *U* test (migration assay).

Based on the above results, it was considered that HSCs in cancer-CM with simultaneous TU-100 treatment did not indicate the cancer cell proliferation- and migration-promoting abilities. To prove this hypothesis, 2 types of HSC-CM were produced. The HSCs were cultured for 24 hours in cancer-CM with or without TU-100 treatment (90, 270, 900). The resulting CM was subsequently exchanged for 2 types of HSCs (which were named aHSC and modified HSC) and obtained aHSC-CM and TU-100 (90, 270, 900) treated HSC-CM (modified HSC-CM) after 24 hours (Supplementary Figure 2). Compared with normally cultured medium, aHSC-CM led to the significantly increased proliferation-activity of colon cancer cells (Figure 2A). Meanwhile, the TU-100 modified HSC-CM (270, 900) significantly suppressed the cancer cell growth and migration compared to the aHSC-CM (Figure 2A–2C). Next, an IL-6 antibody was used to neutralize the IL-6 pathway in the aHSC-CM. IL-6 neutralization significantly cancelled the ability of cancer cells migration and proliferation induced by aHSC-CM, compared with Immunoglobulin G (IgG) as a control (Figure 3A–3C).

### TU-100 directly reduced cancer malignant potential in aHSC-CM

**Figure 4:**
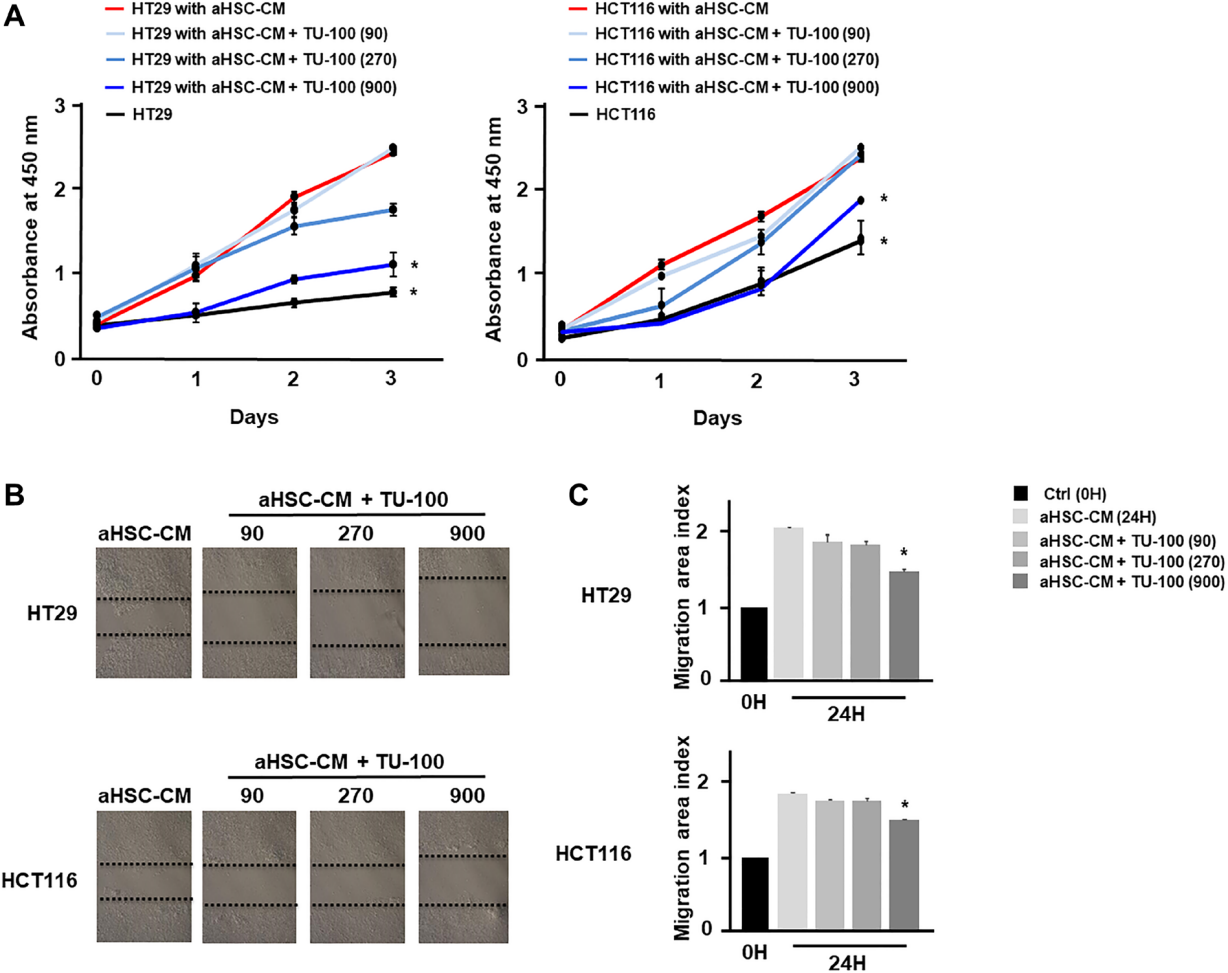
TU-100 directly reduced cancer malignant potential in activated HSC conditioned. Colon cancer cells (HT29 and HCT116) were cultured for 24 hours in aHSC-CM with or without simultaneous TU-100 (90, 270, 900) treatment as described in Supplementary Figure 3. (**A**) Proliferation assay and (**B**, **C**) migration assay of cancer cells cultured in aHSC-CM with or without TU-100 treatment were monitored for 3 days and 24 hours, respectively. (^*^significantly different from aHSC-CM group without TU-100 treatment, *P* < .05, *n* = 4, The one-way ANOVA with Turkey-Kramer’s test (proliferation assay), Mann-Whitney *U* test (migration assay).

The anti-tumor effect of TU-100 has been demonstrated in several cancer cells and mouse models [19, 20]. To investigate the direct anti-tumor effect of TU-100, colon cancer cells were cultured for 24 hours in the presence of aHSC-CM and TU-100 (90, 270, 900) or aHSC-CM alone (Supplementary Figure 3). The cell growth and migration abilities of cancer cells cultured in aHSC-CM and TU-100 (900) treatment were shown to be significantly decreased (Figure 4A–4C).

Japanese pepper and ginseng, components of TU-100 and one of the mechanisms for the direct anti-tumor effect of TU-100, have been reported to promote autophagic cell death in cancer cells [34–39]. Autophagy is initiated by UNC51-like kinase (ULK) 1, Beclin1 and PI3K complexes that promote the formation of an isolation membrane [40]. The isolation membrane gets decorated with microtubule-associated protein 1A/1B-light chain 3 (LC3) which serves as an anchor for recruiting the selected cargo. Increase in the mRNA and protein expression of several autophagy core genes and proteins such as Beclin1 and LC3 often accompany the induction of autophagy [41].

**Figure 5:**
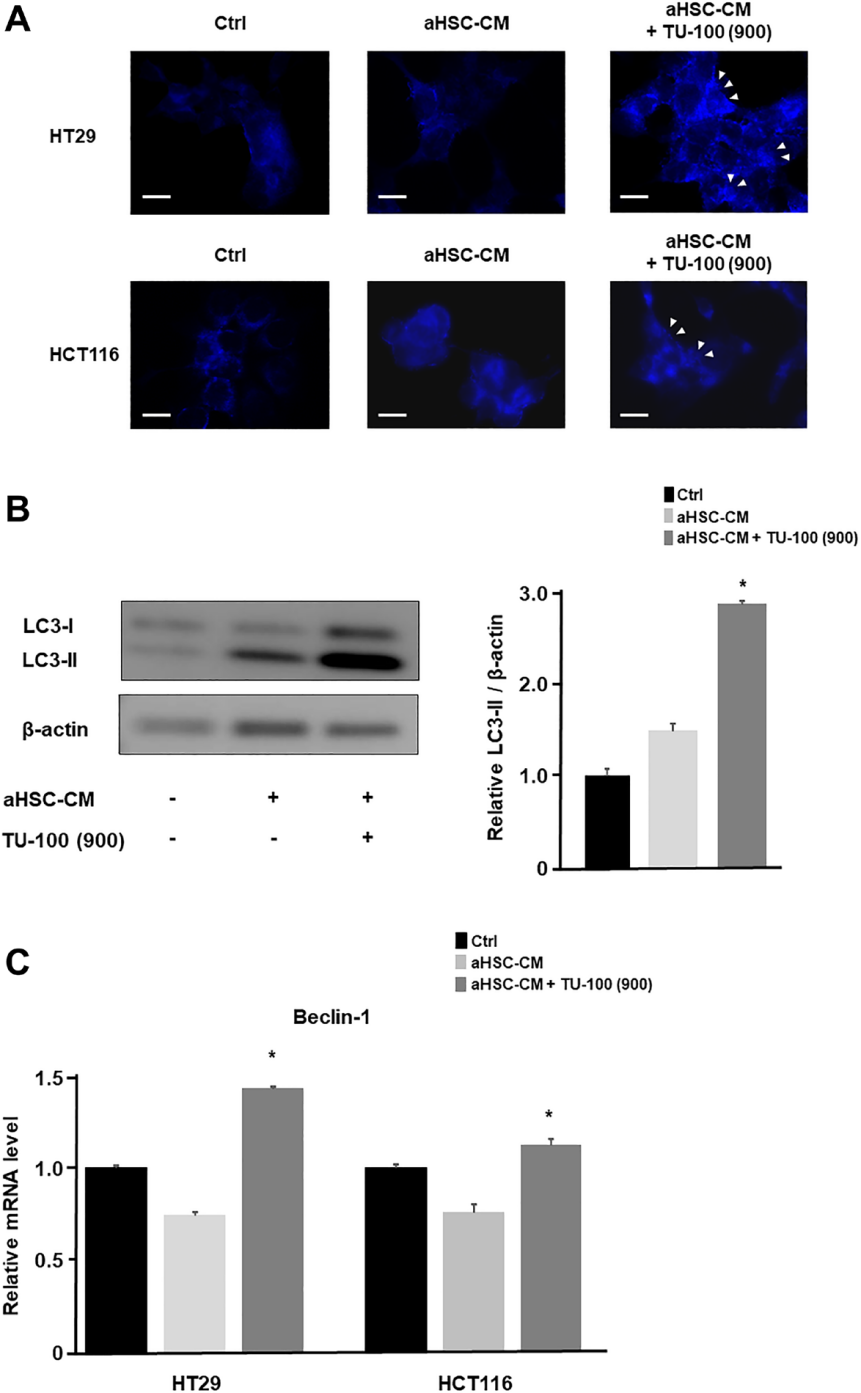
TU-100 induced the autophagic cell death. (**A**) To assess autophagosomes, colon cancer cells (HT29 and HCT116) in aHSC-CM with or without TU-100 (900) treatment were stained with blue dots in autophagic vacuoles (blue) and DAPI (blue). Arrowhead; bright blue dots indicate autophagy. Scale bar, 10 μm. (**B**) Western blotting of LC3-I and LC3-II in HT29 cultured HSC’s supernatants (aHSC-CM) with or without TU-100 (900) treatment. (**C**) Beclin-1 mRNA expressions of cancer cells were detected by PCR analysis. (^*^significantly different from aHSC-CM group without TU-100 treatment, *P* < .05, *n* = 4, Mann-Whitney *U* test).

Therefore, we assessed the confirmation of autophagosomes and conversion of LC3-I to LC3-II in Western blots, besides increased mRNA expression of Beclin1 as indicators of autophagy. We detected the formation of autophagosomes in cancer cells cultured in aHSC-CM with TU-100 (900) treatment (Figure 5A). In addition, we confirmed the conversion of LC3-I to LC3-II in HT29 cultured with TU-100 (900) treatment by Western blotting (Figure 5B) and Beclin-1 mRNA expression was also increased in TU-100 (900) treatment (Figure 5C). Consequently, these results show TU-100 induce the death of autophagic cells.

## DISCUSSION

Among all herbal medicines, some have been reported to have anti-tumor and anti-inflammatory effects [42–44]. TU-100 is one of the herbal medicinal extract preparations available in Japan [10]. Furthermore, TU-100 has been approved as a drug by the US Food and Drug Administration. Our present study revealed that TU-100 inhibited tumor growth of cancer cells by suppressing HSC activation and decreasing IL-6 secretion from HSC, as well as inducing directly the autophagy of cancer cells. This has the potential to lead to clinical application and novel approach to control tumor growth in safety.

In the tumor microenvironment of CRLM, Kupffer cells and HSCs are activated by cancer cells, and those activated stromal cells interact with metastatic cancer cells and induce tumor infiltration [23]. In several reports, IL-6 has been shown to play a key role for promoting the cancer cell malignancy in the tumor microenvironment [45–47]. As we previously reported that activated HSCs promote cancer cell progression through paracrine or autocrine IL-6, it is known that secretion of CAFs and activated HSC regulate downstream pathways and promote tumor growth [48]. To overcome this aspect, we have noticed that TU-100 suppresses HSC activation and liver fibrosis in the common bile duct ligation rat model [29]. This led to focus on the possible anti-tumor effect of TU-100 to cancel the interaction between HSCs and tumor cells, and present studies have shown that TU-100 has inhibited HSC activation promoted by cancer cells, and suppressed the secretion of IL-6 in HSCs. As IL-6 secretion was showed to be most suppressed in the supernatant of HSCs with TU-100 treatment, we confirmed that anti-IL-6 antibody has the effect of tumor regression as well as TU-100. Although these reports supported present studies, VEGF secretion was also suppressed in the supernatant from HSC and the role of VEGF should be validated in future experiment.

Regarding as the mechanism of suppressing HSC activation, we previously reported that the suppression of the toll-like receptor 4 (TLR4) and transforming growth factor β (TGF-β) showed one of the important mechanism related to the pharmacologic effect of TU-100 [29]. In other possibilities, Ginseng contained in TU-100 have reported that liver X receptors (LXRs), nuclear factor erythroid 2-related factor 2 (Nrf2) and janus kinase 2 (Jak2)/ signal transducer and activator of transcription 3 (Stat3) pathways are involved in HSC suppression [49, 50]. Although, it is necessary to continue investigating the mechanism of TU-100 that suppresses HSC activation, this study showed that TU-100 decreased IL-6 secretion from HSCs and inhibited cell migration and proliferation through the suppressing of HSC activation.

Additionally, this study showed the direct anti-tumor effect of TU-100 on cancer cells through autophagy induction. Autophagy degrades the cytoplasmic components by delivering to the lysosomes [51, 52]. Cancer cells depend on autophagy than normal cells because hypoxic situation of the tumor increased the activity of autophagy [53, 54]. Cancer cells exposed to stress maximize the energy production by upregulating autophagy and induced cell death by excessive activation of autophagy [55]. Although it is still unknown the mechanism of TU-100 to induce autophagic cell death, previous reports demonstrated that ginseng, ginger, and Japanese pepper which are contained in TU-100 induced programmed cell death like autophagy and apoptosis [34–39, 56, 57]. It was reported that Japanese pepper promotes cancer cell death by inducing autophagy [36, 57]. Ginseng metabolites which is formed in bacterial glycosidases and glucosidases have important part for an anti-tumor activity [58]. Ginseng triterpendanmarandine senoside Rb1 is deglycosylated and easily absorbed, producing compound K with anti-tumor activities such as cancer cell autophagy [59, 60]. Also, ginger and ginseng have been reported to block Akt and extracellular-regulated kinase (ERK) signaling [20]. In addition, TU-100 reduced the expression of reactive oxygen species (ROS), β-catenin and Hes-1 which plays an important role in various cellular responses, like autophagy [20]. Although these reports have favoured our results, the mechanism of cell death in TU-100 should be performed in future experiment.

In summary, this study has shown that TU-100 inhibited the malignant potential of cancer cells by both suppressing HSC activation and secretion of IL-6 from HSCs, and inducing directly the autophagy of cancer cells. These results demonstrate that TU-100 could be one of the promising therapeutic agent for the treatment of CRLM.

## MATERIALS AND METHODS

### Cell culture

The human colorectal cancer cell lines (HCT116 and HT29) were originally obtained from the American Type Culture Collection (ATCC, Manassas, VA, USA). The human HSC line (LX2) was obtained from Sigma-Aldrich (St Louis, MO, USA). Cancer cell lines were maintained in McCoy’s 5A Medium (Life Technologies Ltd. Tokyo, Japan) containing 10% fetal bovine serum (FBS) (Life Technologies Ltd.) and incubated at 37°C in a humidified 5% CO2. The HSCs were maintained in Dulbecco’s Modified Eagle Medium (DMEM) (Life Technologies Ltd.) containing 10% FBS and incubated at 37°C in a humidified 5% CO2.

### Cell preparation with conditioned medium (CM)

Cancer cells (3.0 × 10^6^ cells) were maintained in McCoy medium with 1% FBS for 24 hours in a 10 cm dish. Then, we discarded the supernatant and cancer cells were cultured in the medium of HSCs (3.0 × 10^5^ cells) for 24 hours. After that, the procedure for a FBS-free medium was cultured. After 24 hours, the supernatant was collected, centrifuged at 450× g for 5 minutes and filtrated using a 0.2 μm filter [61]. The CM was added without any additional FBS. For the IL-6 of CM neutralization, IL-6 antibody (7270-IL; R&D Systems, Minneapolis, MN) was added to the CM with a concentration of 0.2 μg/mL and incubated for 1 hour.

### Reagents

TU-100 was obtained from Tsumura & Co (Tsumura Daikenchuto Extract Granules; Tsumura & Co, Tokyo, Japan). As previously noted, TU-100 is an extract powder from a mixture of Japanese pepper (Zanthoxylum fruit), processed ginger (Zingiberis Siccatum Rhizoma), and ginseng (Ginseng radix) [10, 16, 62]. The each concentrations of TU-100 (90, 270, and 900 μg/mL) were added to the medium using the previously described method [15, 17, 29, 63].

### Cell proliferation assay

Cell proliferation assays were performed with a Cell Counting Kit-8 (CCK8) (Dojindo Molecular Technologies, Inc. Kumamoto, Japan). Cancer cells were plated in 24 well plates and incubated with 10% CCK8-CM for 2 hours. The cells were measured by using the plate reader (SpectraMax i3, Molecular Devices, Tokyo, Japan) at the absorbance 450 nm. We used IgG (Cat#1–001-A, R&D Systems) as a control.

### Scratch assays

For scratch assays, cancer cells (3.0 × 10^6^ cells) were plated in 6 cm dishes. The medium of cancer cells (3.0 × 10^6^ cells) was transferred to activated HSCs conditioned medium (aHSC-CM) or DMEM medium (control) and cultured for 24 hours. When cells are confluent, a plastic pipette tip was used to make 1-mm-wide area. After culturing for 24 hours in a medium with 1% FBS, wound closure was monitored by microscope under phase to measure the wound area [64].

### Enzyme-linked immunosorbent assay

The levels of IL-6 and VEGF were detected by using IL-6 and a VEGF Quantikine ELISA kit (R&D Systems). The wavelength was measured at 450 nm using a plate reader (SpectraMax i3; Molecular Devices) as a detection of emission at a wavelength of 540 nm.

### Polymerase chain reaction analysis

Total RNA was extracted from each cell using the RNeasy Mini Kit (Qiagen, Hilden, Germany). Synthesis of complementary DNA was conducted using the reverse transcription kit (Applied Biosystems, Thermo Fisher Scientific Inc., Waltham, MA, USA). The primer sequences from TaqMan gene expression assays (assay identification number) for the target genes used in the present study are shown as followed: α-SMA (Hs05005341_m1); IL-6 (Hs00174131_m1) and Beclin-1 (Hs01061917_g1). The relative abundance of target transcripts was evaluated and normalized to the expression of Glyceraldehyde-3-Phosphate Dehydrogenase (GAPDH) (4352339E) as an internal control. Real-time quantitative reverse transcription polymerase chain reaction (RT-qPCR) analysis was performed on the StepOnePlus Real-Time PCR System (Applied Biosystems).

### Cytokine array

Supernatants of the aHSCs with or without TU-100 (900 μg/mL) treatment were collected, and the debris was eliminated through a 0.2 μm filter. Cytokine detection in the supernatants was performed using a Proteome Profiler Human Cytokine Array Kit (ARY005B; R&D Systems). Array membranes were spotted by each capture antibodies and incubated with samples overnight at 4°C. After incubation, the membranes were washed three times, and incubated with streptavidin- horseradish peroxidase (HRP)-coupled antibody at room temperature for 30 minutes. Then, the membranes were incubated with Chemiluminescent reagents for 1 minute, and the intensity of the chemiluminescent reaction on the membranes was detected by chemiluminescence (GE Healthcare, Little Chalfort, UK). The relative level of cytokine was determined by measuring the density of each spot for comparison with those variables of negative and positive standards.

### Detection of autophagosomes

Autophagosomes were detected by using a Cell Meter™ Autophagy Assay kit Blue (AAT Bioquest, Inc., Sunnyvale, CA, USA) in HT-29 and HCT-116 with TU-100 (900 μg/mL). Treated with TU-100 and negative control cells were cultured with Autophagy Blue™ working solution and incubated at 37°C for 1 hour. Cancer cells were washed for three times, and then examined under a BZ-x700 fluorescence microscope (Keyence, Osaka, Japan).

### Western blotting

Cells were collected and lysed in RIPA buffer (Thermo Fisher Scientific Inc.) supplemented with protease inhibitor cocktail (Sigma-Aldrich) and the PhosSTOP phosphatase inhibitor cocktail (Roche, Tokyo, Japan). The protein concentration of the samples was determined with the BCA assay kit (23225; Thermo Fisher Scientific Inc.). An equal amount of extracted proteins was separated on 10% SDS-PAGE Gel, and transferred onto PVDF membrane (162-0177; Bio-Rad Inc., Hercules, CA, USA) [65]. The membranes were blocked and incubated in the indicated primary antibody, followed by the appropriate HRP-conjugated secondary antibody. The bands detected by chemiluminescence (Thermo Fisher Scientific Inc.). The primary antibodies used are; LC3 (ab51520, Abcam, Cambridge, MA, USA), and β-actin (Sigma Chemical, St Louis, MO, USA).

### Statistics

The statistical analyses of experiments were performed using the Mann-Whitney *U* test between 2 groups or one-way ANOVA with the Turkey-Kramer’s test between 3 or more groups. Data analysis was performed with JMP V 13 statistical software (SAS Campus Drive, Cary, NC). All data were expressed as the mean ± SD. Statistical differences were considered as significant with a *P* value < 0.05.

## SUPPLEMENTARY MATERIALS



## Abbreviations

α-SMA: α-smooth muscle actin
CAF: cancer-associated fibroblast
CCK8: cell-counting kit-8
Colla1: collagen type 1
CRC: colorectal cancer
CRLM: colorectal liver metastasis
CM: conditioned medium
DMEM: Dulbecco›s Modified Eagle Medium
ERK: extracellular-regulated kinase
FBS: fetal bovine serum
GAPDH: gyceraldehyde-3-Phosphate Dehydrogenase
HSCs: hepatic stellate cells
IgG: immunoglobulin G
IL-6: interleukin-6
Jak2: janus kinase 2
LXRs: liver X receptors
mRNA: messenger RNA
LC3: microtubule-associated protein 1A/1B-light chain 3
Nrf2: nuclear factor erythroid 2-related factor 2
PD-L1: programmed cell death ligand 1
ROS: reactive oxygen species
Stat3: signal transducer and activator of transcription 3
Timp1: tissue inhibitor of metalloproteinase 1
TLR4: toll-like receptor 4
TGF-β: transforming growth factor β
Daikenchuto: TU-100
ULK: UNC51-like kinase
VEGF: vascular endothelial growth factor
