# Supplementary material for: The inhibitory effect of TU-100 on hepatic stellate cell activation in the tumor microenvironment
Source: Oncotarget. 2020 Dec 8;11(49):4593–604. doi: 10.18632/oncotarget.27835 (PMC7733620; doi:10.18632/oncotarget.27835)
Supplement: Supplementary file 1 [file oncotarget-11-4593-s001.pdf]

The inhibitory effect of TU-100 on hepatic stellate cell activation in the tumor microenvironment

SUPPLEMENTARY MATERIALS

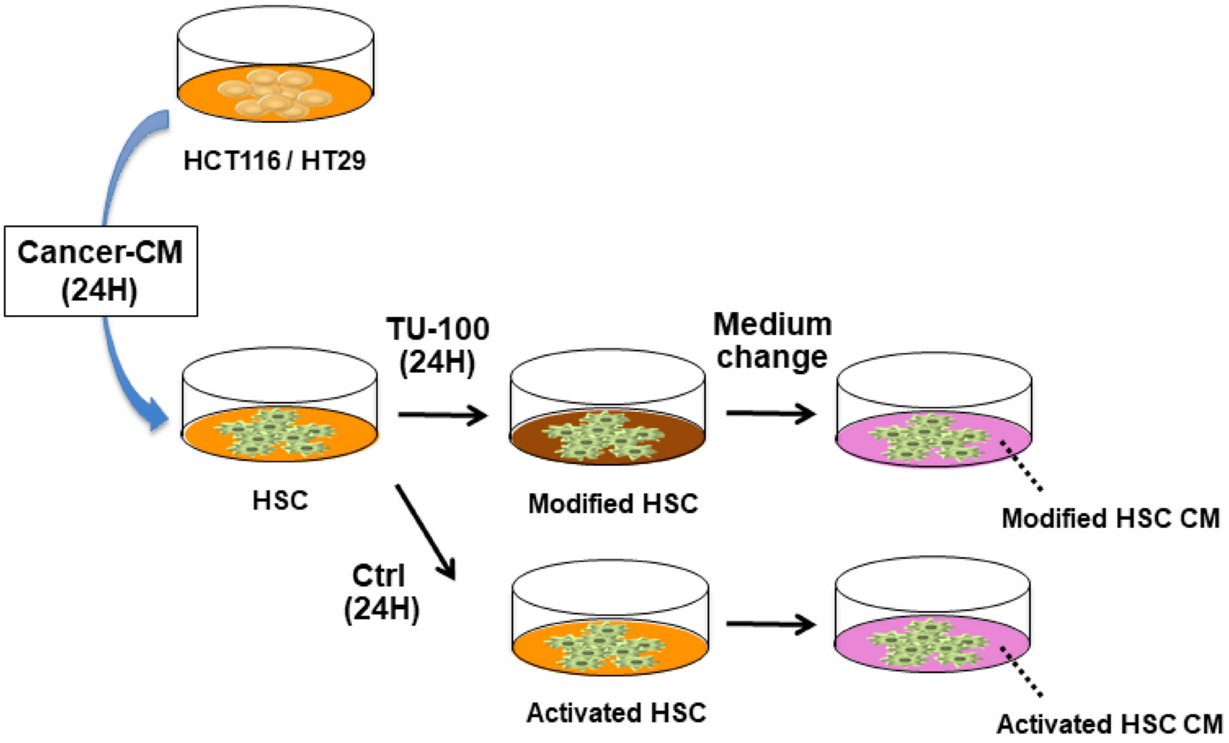

Supplementary Figure 1: HSCs were stimulated with cancer-conditioned medium (cancer-CM) derived from 24 hours cancer cells (HT29 and HCT116) culture. The HSCs were cultured for 24 hours in cancer-CM with or without simultaneous TU-100 (90, 270, 900) treatment. After that, the new conditioned medium was exchanged for 2 types of HSCs, which were named as activated HSCs (aHSCs) and modified HSCs.

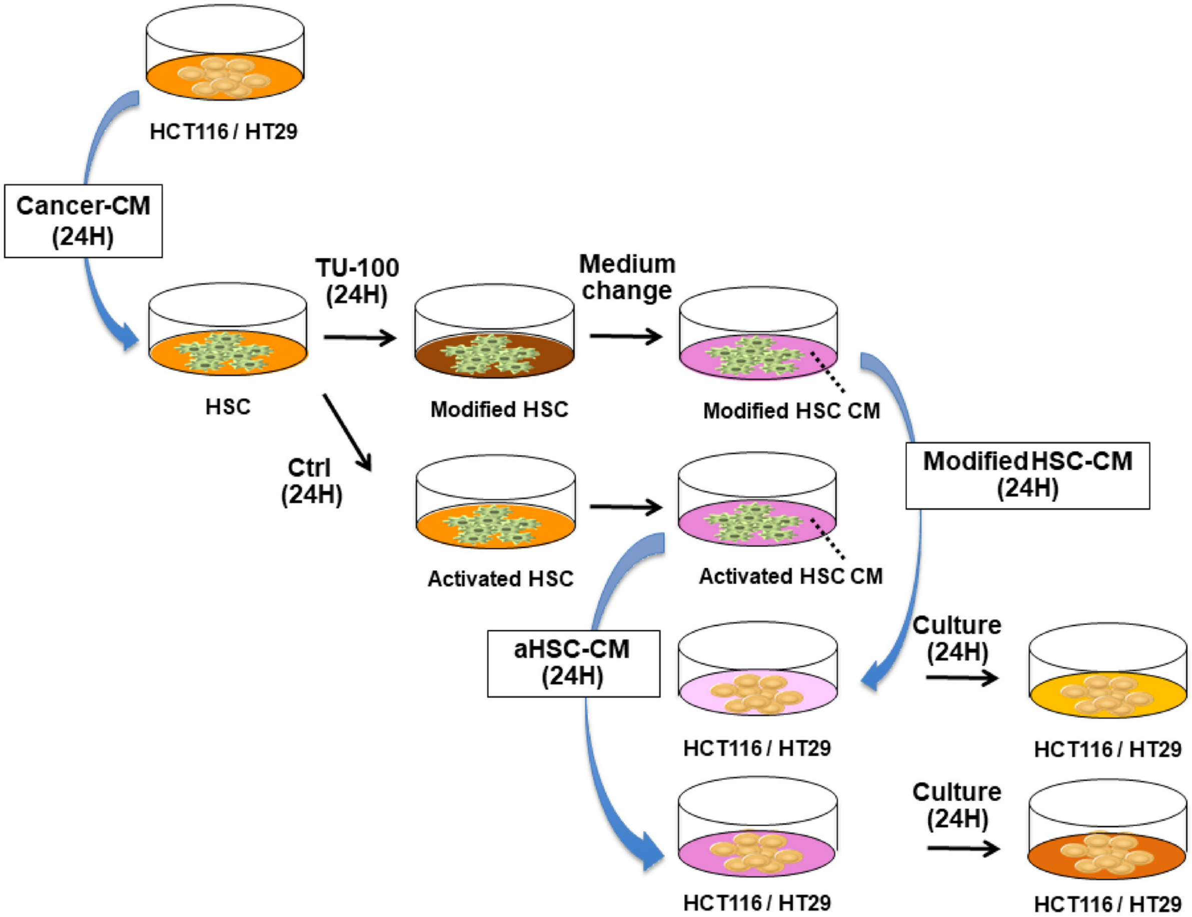

Supplementary Figure 2: The HSCs were cultured for 24 hours in cancer-CM with or without simultaneous TU-100 treatment (90, 270, 900). After that, the new conditioned medium was exchanged for 2 types of HSCs (which were named as activated HSCs and modified HSCs) and obtained activated HSC-conditioned medium (aHSC-CM) and TU-100 treated HSC-conditioned medium (modified HSC-CM) after 24 hours. Cancer cells (HT29 and HCT116) were cultured for 24 hours with aHSC-CM or modified HSC-CM.

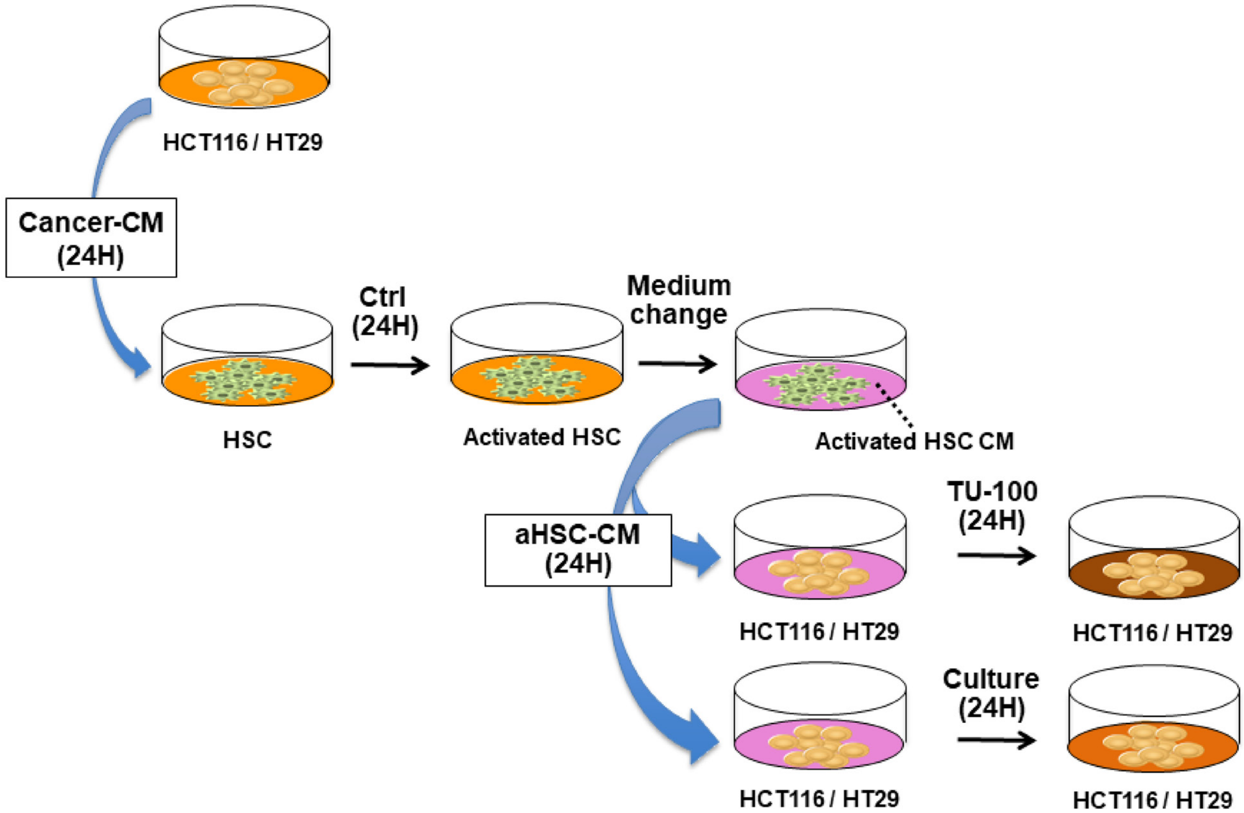

Supplementary Figure 3: The HSCs were cultured for 24 hours in cancer-CM. After that, the new conditioned medium was exchanged for activated HSCs (aHSCs) and aHSC-CM was obtained after 24 hours. Cancer cells (HT29 and HCT116) were cultured for 24 hours in aHSC-CM with or without simultaneous TU-100 (90, 270, 900) treatment.
